# Root canal conicity of primary maxillary molars and its relationship with different rotational systems

**DOI:** 10.1007/s40368-025-01075-w

**Published:** 2025-07-11

**Authors:** Jesús Miguel Ticona-Flores, Nuria Esther Gallardo-López, Montserrat Diéguez-Pérez

**Affiliations:** 1 Complutense University of Madrid, Madrid, Spain; 2 European University of Madrid, Madrid, Spain

**Keywords:** Rotary files, Endodontics, Primary teeth, Pulpectomy

## Abstract

**Aim:**

To determine the root canal taper of primary maxillary molars and the degree of compatibility of various rsotary systems concerning root anatomy.

**Materials and methods:**

This in vitro study collected donated first and second primary molars (1 M and 2 M) with mesio-buccal (MB), disto-buccal (DB) and palatal (P) roots canal without physiological resorption, type I according to Vertucci, and root length greater than 4 mm. The teeth were mounted in silicone blocks and scanned with tomographic equipment from which images were reconstructed and analysed with the 3D-Slicer® programme, allowing for the measurement of the diameters of the root canals and the calculation of their tapers. The tapers were compared with the characteristics of the rotary systems: Endogal®, Protaper universal®, Mtwo® and Protaper Next®. MANOVA and interclass correlation coefficient (ICC) tests were used for statistical analysis.

**Results:**

After the analysis of 130 root canals, a mean increase in taper in the buccal-palatal (BP) direction was observed in the 2 M (MB:16.7%; DB:16.23%; P:8.86%) and the 1 M (MB: 9.75%; DB: 11.30%; P: 2.26%). In the mesiodistal (MD) direction, the 1 M exhibited an average taper of MB:6.95%, DB:4.67%, P:12.74% and in the 2 M, an average taper of 4.67% for the MB canal; 6.60%; 20.14% for DB and P canals, respectively.

**Conclusion:**

The rotary files that presented the best adaptation to the diameter and taper of the root canal were Endogal® and ProTaper Universal® systems.

## Introduction

Pulpectomy is a treatment option treatment for preserving primary teeth with irreversible inflammation or necrotic pulp pathology. The success of pulpectomy depends on multiple factors, including optimal biomechanical preparation, irrigation, effective disinfection, obturation, and tooth restoration (Chauhan et al. 2019).

The mechanical preparation of root canals is influenced by the significant obstacle caused by the anatomical complexity of primary molar root canals (Morankar et al. 2018).

Currently in paediatric dentistry there is no standardised protocol regarding the application of rotary files nor studies on how anatomical irregularities of the primary dentition can diminish their effectiveness (Boutsioukis et al. 2010; Chauhan et al. 2019).

The use of rotary systems in primary teeth has been reported in several studies, primarily involving systems such as ProTaper Universal® (Dentsply Maillefer, Ballaigues, Switzerland) and Mtwo®(VDW GmbH, Munich, Germany), which were originally designed for use in permanent teeth. These systems, reported to have efficiency in canal shaping, have become widely adopted in paediatric endodontics due to their commercial availability (ElAyouti et al. 2008). However, these systems have limitations when applied to primary teeth, including an increased risk of over-instrumentation and potential damage to periapical structures due to their greater taper and length. More recent systems, such as ProTaper Next® (Dentsply Sirona, Ballaigues, Switzerland) and EndoGal Kids®, (Endogal, Lugo, Spain) are manufactured from thermally treated nickel-titanium, providing enhanced flexibility and resistance to cyclic fatigue, thereby improving performance in curved canals (Faus-Llácer et al. 2022). The Endogal Kids® system is reported to have been designed specifically for the instrumentation of primary teeth, it features reduced lengths (16 mm) and diameters tailored to primary root canal anatomy. It exhibits a controlled taper ranging from 4 to 6%, promoting more conservative canal preparation and reducing the risk of perforations or root weakening (Faus-Llácer et al. 2022). In comparison, the ProTaper Universal® system typically has tapers varying from 2 to 8%, depending on the file size and sequence, often with a greater taper at the coronal portion. In contrast, the Mtwo® system offers a consistent taper of generally around 4%, which may be less aggressive than that of the ProTaper Universal® system. Therefore, the more moderate and controlled taper of Endogal Kids® helps preserve tooth structure better than some conventional rotary systems designed for permanent teeth . Furthermore, its triangular cross-sectional design, shared with the ProTaper Universal® system, facilitates better centring within the canal and greater cutting efficiency without compromising the original anatomy (Juliet et al. 2020).

The use of an inappropriate file can lead to a fracture of the instrument due to cyclical fatigue (Din et al. 2013) or torsional failure or inadequate preparation of the root canal, as occurs in oval root canals, (ElAyouti et al. 2008) favouring inefficient disinfection, which, depending on the root portion, could constitute inadequate instrumentation in a high proportion of cases (42–53%) (Veloso-Carvalho de Oliveira et al. 2014). Although the scientific literature reports such inadequate canal shaping throughout the root canal of primary teeth, its causal factors need to be clarified, as it is related to the inadequate adaptation of rotary instruments and the anatomical characteristics concerning the conicity of the root canal (ElAyouti et al. 2008; Veloso-Carvalho de Oliveira et al. 2014). Hence, it is relevant to investigate the anatomical conformation of root canals and its correlation with the instrumentation systems on the market (Neboda et al. 2018). Thanks to the advancement of three-dimensional technology with CBCT and micro-CT records in in vitro studies (Neboda et al. 2018), it is possible to reconstruct and analyse DICOM images into three-dimensional images (Fedorov et al. 2012; Ticona-Flores and Diéguez-Pérez 2022), allowing the study of dimensional differences of the different diameters, dentine thickness and root curvatures. (Veloso-Carvalho de Oliveira et al. 2014).

It was hypothesised that the Endogal®, due to its paediatric-oriented design, would adapt better to undertaking pulpectomies on primary teeth.

Based on the above, the purpose was to determine the taper of the primary maxillary molar canals and their modification with the Endogal®, Protaper universal®, Mtwo® and Protaper Next® rotary systems.

## Materials and methods

An in vitro, observational, descriptive, and comparative study was designed, which was approved by the Ethics Committee of the Hospital Clínico San Carlos with internal code 21/375E.

Primary maxillary first molars (1 M) and second molars (2 M) were collected from donated teeth with the approval and signing of the informed consent of the parents of the child patients. This donation occurred both in public and private dental clinics. The indication for tooth extraction was due to clinical reasons such as orthodontic reasons or delayed eruption.

### Eligibility criteria and sample size

The study unit was all root portions without resorption and a complete apex, including the mesio-buccal (MB), disto-buccal (DB), and palatal roots. All teeth had Type I root canal anatomy according to the classification described by Vertucci et al. (Vertucci 1978). The minimum root length was 4 mm. Roots presenting any signs of external or internal pathological or physiological resorption that affected the apical foramen or pulp treatment were excluded.

The sample size was calculated using the G*Power® statistical programme. The diameters of the cervical third of each canal were taken as the reference point: M: 1.18 ± 0.6; D: 1.17 ± 0.25 and P: 2.56 ± 0.37 (Gaurav et al. 2013). Additionally, a power of 80% and a significance level of p < 0.05 were established, estimating that a sample size of at least 12 canals is required for each group. The study included six groups in total, which were classified according to the type of primary molar—first or second, and the canal type: mesio-buccal (MB), disto-buccal (DB), or palatal (P).

### Storage samples and formation Group

All treating dentists extracted and initially stored the teeth in isotonic saline solution (0.9% NaCl), which was provided in advance to ensure consistent storage conditions. Subsequently, the researcher collected them weekly and took them to the laboratory where they were debrided using Dentasept® Tri Enzymatic soap (DÜRR DENTAL, Bietigheim-Bissingen, Germany) a commercial enzymatic detergent containing protease, lipase and amylase, designed for the removal of organic residues. The teeth were then disinfected by immersion in 3% sodium hypochlorite for one week. The final storage was performed in sterile containers with sodium chloride 0.9% and kept at 4 ºC until use. The average storage period until analysis was 45 days.

The roots of the teeth were distributed into six groups:

- Groups 1 and 2: mesio-buccal roots (MB) of the 1 M and 2 M, respectively.
- Groups 3 and 4: disto-buccal roots (DB) of the 1 M and 2 M, respectively.
- Groups 5 and 6: palatal roots (P) of the 1 M and 2 M, respectively.

### CBCT image acquisition and 3D reconstruction

The roots were invested into addition silicone material blocks (polyvinyl siloxane, PVS) (V-Posil Putty Fast, VOCO®, Cuxhaven, Germany) for irradiation, with DICOM images with a slice thickness of 0.75 µm obtained using a CS 8100® tomograph (Carestream Dental, GA, USA) with a voltage of 90 kV, a tube current of 15 mA, exposure time of 7–15 s, voxel size of 75 µm, and a field of view (FOV) of 4 mm × 4 cm.

With the images and the 3D-Slicer® programme the 3D reconstruction of the root portions was performed (Fedorov et al. 2012). The "Threshold" tool allowed the calculation of the Hounsfield Units (HU) of the different structures. A range of −1000 to 250 HU was established for the root canals and 1294.4 ± 183.58 HU for the root  portions.

### Calculation of the taper of root canals

The root canal length was measured from the apical constriction to its most superior sector with the 3D-Slicer® programme using the "Line" Tool. The mean lengths helped standardise the number of transverse measurements (diameters) for each canal root (MB, DB or P). Each canal was then proportionally divided based on its relative length compared to the mean length of canals in the same group (MB, DB, or P), using the following formula to obtain the distance between sections (DBS).

$$DBS=\frac{\text{Root length} }{\text{Mean root length }\left(\text{M},\text{ D or P}\right)}$$

**Fig. 1:**
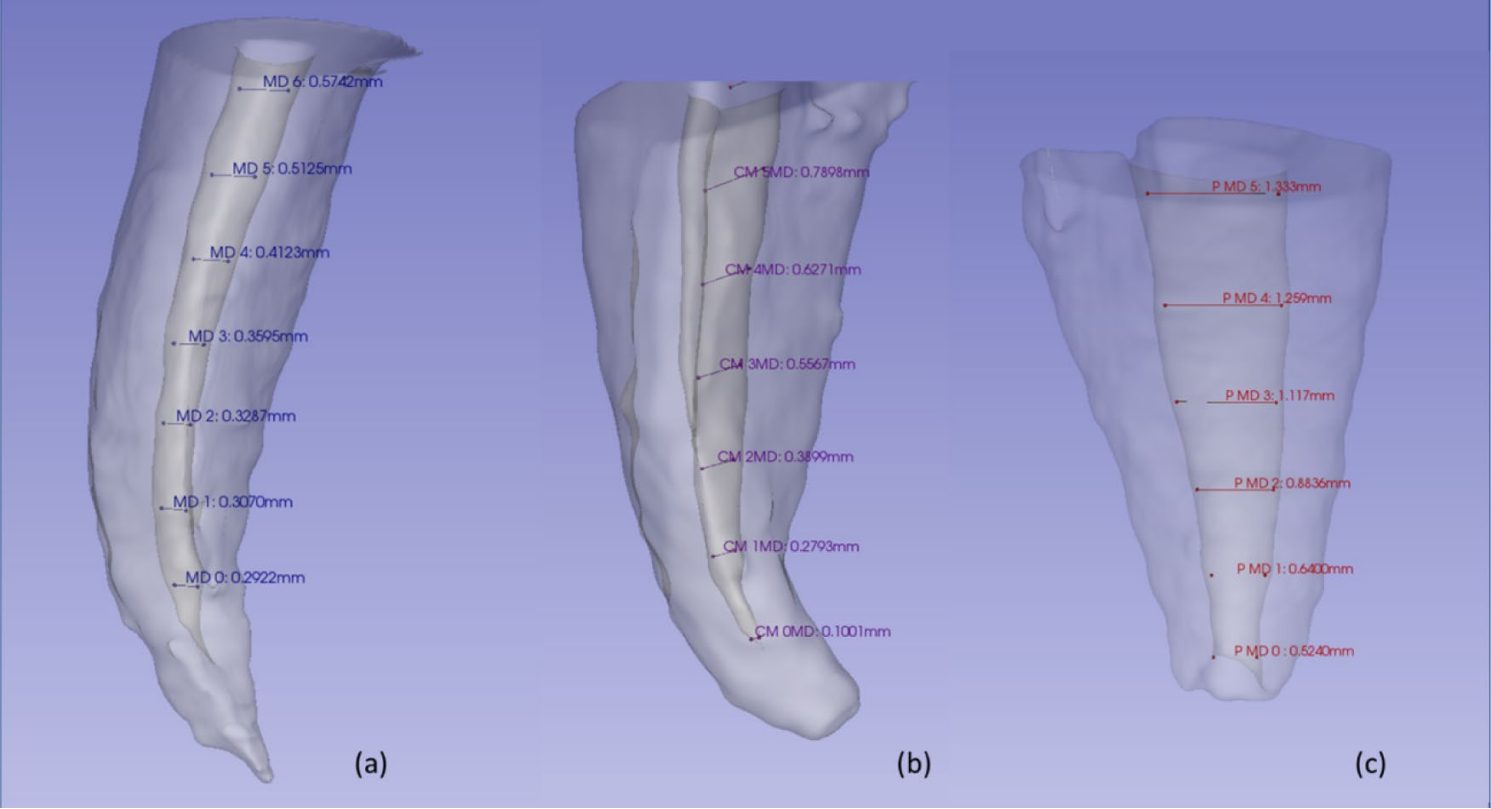
Representation of taking measurements in all three-dimensional reconstructions. **a** Mesio-buccal root; **b** Disto-buccal Root); **c** Palatal root

Next, all the diameters along the canal in the bucco-palatal (BP) and mesiodistal (MD) directions were calculated, as shown in Fig. 1.

The bucco-palatal (BP) diameter refers to the horizontal measurement across the root canal, extending from the buccal to the palatal wall of the root canal, whereas the mesiodistal (MD) diameter corresponds to the measurement from the mesial (proximal to the midline of the dental arch) to the distal (away from the midline) wall of the root canal.

The apical constriction was considered the initial measurement (D0) (Kayabasi and Oznurhan 2020). Subsequently and equidistantly, the remaining measurements were obtained according to the result obtained by the DBS of each root canal.

The taper expressed in percentage (%) was calculated for each segment, according to the following formula (Torres-Ramos et al. 2020):

$$Taper= \frac{{D}_{L}-{D}_{s}}{d}$$

D_L_ and D_s_ were the longest and shortest diameters recorded, respectively. Furthermore, the d value corresponded to the distance between both diameters.

### Canals and files comparison

The MD and BP diameters were measured from D0 with an interval of 1 mm up to the cervical third regardless of the total length of the canal. These measurements were compared with the diameters of the files of the Endogal®, Protaper universal®, Mtwo® and Protaper Next® systems. (Table 1).

**Table 1:** Diameter per millimetre of each instrument

mm	Endogal® (mm)				Protaper universal® (mm)			Mtwo® (mm)				Protaper Next® (mm)	
	EK1 (4%)	EK2 (6%)	EK3 (4%)	EK4 (4%)	S2 (variable)	F1 (~ 7%)	F2 (~ 8%)	0.15 (5%)	0.20 (6%)	0.25 (6%)	0.30 (4%)	X1 (~ 6%)	X2 (~ 6%)
0	0.25	0.25	0.3	0.4	0.2	0.2	0.25	0.15	0.2	0.25	0.3	0.17	0.25
1	0.29	0.31	0.34	0.44	0.22	0.28	0.32	0.2	0.26	0.31	0.33	0.21	0.31
2	0.33	0.37	0.38	0.48	0.25	0.36	0.39	0.25	0.32	0.37	0.36	0.26	0.37
3	0.37	0.43	0.42	0.52	0.29	0.44	0.46	0.3	0.38	0.43	0.39	0.31	0.43
4	0.41	0.49	0.46	0.56	0.335	0.5	0.515	0.35	0.44	0.49	0.42	0.375	0.5
5	0.45	0.55	0.5	0.6	0.385	0.555	0.57	0.4	0.5	0.55	0.45	0.44	0.57
6	0.49	0.61	0.54	0.64	0.445	0.61	0.625	0.45	0.56	0.61	0.48	0.505	0.64
7	0.53	0.67	0.58	0.68	0.515	0.665	0.68	0.5	0.62	0.67	0.51	0.58	0.71
8	0.57	0.73	0.62	0.72	0.595	0.72	0.735	0.55	0.68	0.73	0.54	0.655	0.78
9	0.61	0.79	0.66	0.76	0.685	0.775	0.79	0.6	0.74	0.79	0.57	0.73	0.85
10	0.65	0.85	0.7	0.8	0.785	0.83	0.845	0.65	0.8	0.85	0.6	0.79	0.92

The diameters of the rotary files were obtained from each manufacturer's technical specifications

Diameters expressed in mm

The percentage values (e.g., 4%, 6%) indicate the taper of each instrument. The symbol " ~ " denotes an approximate taper, as certain instruments (e.g., ProTaper Universal® F1 and F2) have variable taper along their active length. Approximate taper values are based on the manufacturer's provided specifications

### Calibration, internal validity

One researcher was instructed in using the *3D-Slicer* program (Fedorov et al. 2012) by a faculty professor, unrelated to the research, who specialises in radiology, with extensive practical knowledge in medical imaging and 3D analysis and experienced in the use of the 3D-Slicer software. A pilot test was carried out to train the principal investigator and verify the reliability and reproducibility of the study. The intraclass correlation coefficient statistical test evaluated the intra-observer agreement obtained a result of 0.95 (95% CI 0.91–0.97), which is considered is excellent according to the interpretation by Koo (Koo and Li. 2016).

### Statistical analysis

The statistical analysis was carried out using IBM® SPSS® Statistics for Windows, Version 26.0 (IBM Corp., Armonk, NY, USA). The qualitative variables were described by means and standard deviation (SD). After checking the normality (Shapiro–Wilk BP: p = 0.276 and MD: p = 0.249) and homoscedasticity (Levene BP: p = 0.209 and MD: p = 0.087) of the sample, the MANOVA test was applied to assess the increase in conicity along the root canals. In addition, the Huynh–Feldt test was applied to corroborate the sphericity criteria (W Mauchly p < 0.05), whilst the comparison between different roots was analysed using the Bonferroni test. The interclass correlation coefficient test was used to assess the compatibility of the different rotary systems with root canals, and the ANOVA test was used to determine statistical significance.

A bilateral hypothesis was established, with a power of 80% and a statistical significance level of p < 0.05.

## Results

### Sample distribution

**Fig. 2:**
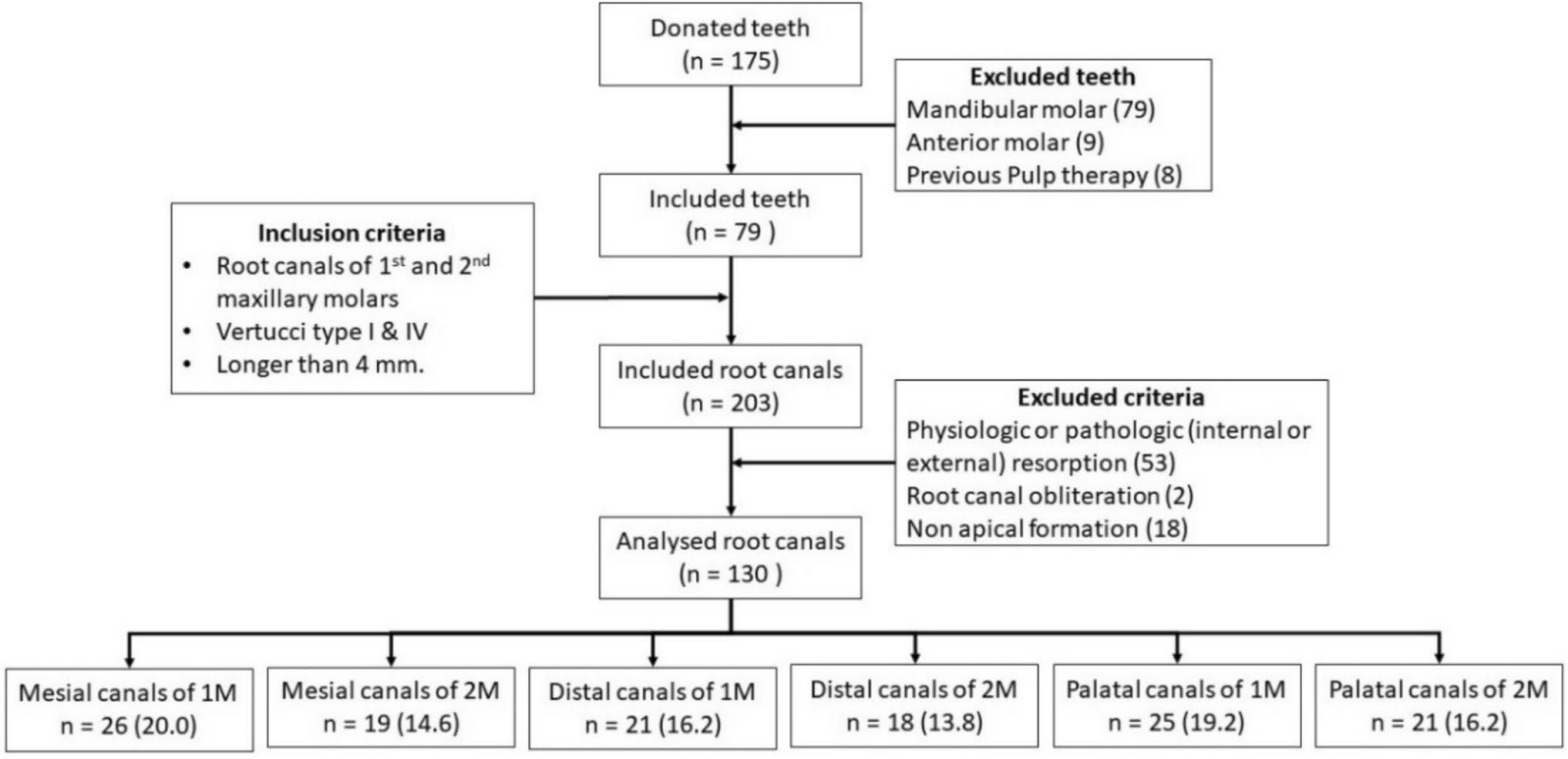
Sample collection flowchart

From a total of 175 teeth and after applying the selection criteria, 79 teeth and 130 root canals were analysed, as described in Fig. 2. The mean length of the root canals in the 1 M was 6.57 ± 1.18 mm for the MB root, 5.74 ± 1.25 mm for the DB root; and 5.94 ± 0.99 mm for the palatal root. In the 2 M, the mean length of the root canals was 7.31 ± 1.02 mm for the MB root; 6.61 ± 1.06 mm for the DB root, and 6.34 ± 0.97 mm for the palatal root. Therefore, seven measurements were possible in the corresponding root canals MB and six in root canals DB and P.

### Taper of root canals

**Fig. 3:**
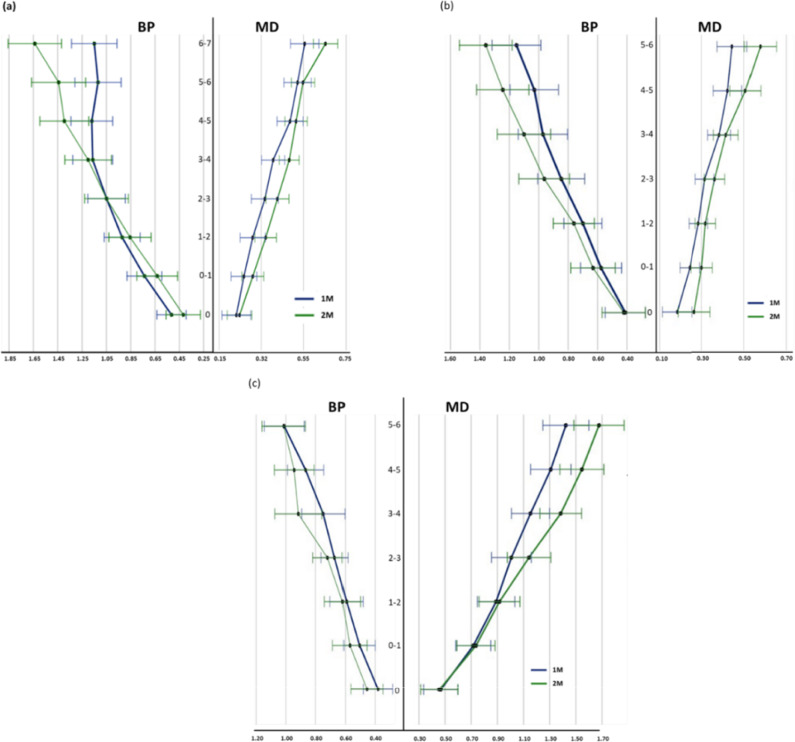
Increase in diameter (mm) per segment analysed. **a** mesio-buccal root; **b** disto-buccal root; **c** Palatal root. BP: Bucco-palatal direction; MD: Mesio-distal direction

The mean diameter at D0 for the 1 M in the BP direction was 0.51 ± 0.29 for the MB root; 0.41 ± 0.29 for the DB root; and 0.38 ± 0.25 for the palatal root. In the MD direction, it was 0.25 ± 0.12 for the MB root; 0.18 ± 0.06 for the DB root; and 0.38 ± 0.25 for the palatal root. Similarly, the mean in D0 for 2 M in the BP direction was 0.42 ± 0.33 for the MB root; 0.42 ± 0.2 for the DB root; and 0.46 ± 0.24 for the palatal root; in the MD direction it was 0.23 ± 0.18 for the MB root; 0.26 ± 0.12 for the DB root; 0.45 ± 0.24 for the palatal root. The change in diameters from D0 to the most cervical portion is presented in Fig. 3, and the change in taper from D0 to the most cervical portion is presented in Table 2.

**Table 2:** Taper of root canals of first and second primary molars

Segment	1 M						2 M					
	MB		DB		P		MB		DB		P	
	Mean %	SD	Mean %	SD	Mean%	SD	Mean %	SD	Mean %	SD	Mean %	SD
BP												
0–1	17.1	7.87	13.54	15.47	10.92	11.32	15.7	11.37	13.52	15.62	10.84	13.74
1–2	16.29	8.66	9.16	13.68	9.96	10.9	23.13	21.06	14.34	13.3	5.92	15.54
2–3	12.34	8.2	13.51	13.73	8.1	11.66	19.6	11.66	23.69	23.74	10.94	9.15
3–4	14.17	20.39	14.49	16.44	7.59	20.72	15.05	17.77	15.61	14.96	16.69	23.82
4–5	6.84	12.37	5.56	19.45	12.49	10.72	18.48	20.59	15.87	18.04	5.62	12.08
5–6	-3.15	14.26	11.54	12.46	6.52	9.19	5.75	20.34	14.33	48.7	3.14	19.11
6–7	4.68	19.08					19.18	18.93				
MD												
0–1	5.19	4.92	5.51	6.68	21.2	16.14	3.46	6.25	8.08	9.33	23.3	12.92
1–2	7.46	7.78	4.2	6.91	12.17	7.53	4.19	5.24	1.89	6.02	20.39	18.84
2–3	6.75	6.18	3.43	7.32	8.43	12.68	6.24	4.86	4.96	8.24	25.45	15.59
3–4	7.04	8.68	6.46	7.32	14.07	19.58	3.22	8.1	5.89	7.71	24.96	21.58
4–5	4.45	9.72	5.46	8.42	12.58	7.84	8.71	12.45	10.02	7.94	17.45	24.19
5–6	3.97	10.32	2.93	10.91	7.98	10.96	3.34	9.78	8.76	16.34	9.26	17.63
6–7	10.31	8.32					3.53	17.15				

Conicity expressed in %

1 M: 1st primary molar; 2 M: 2nd primary molar

MB Mesio-buccal canal; DB: disto-buccal canal; P: Palatal canal

BP: Bucco-palatal diameter; MD: mesio-distal diameter

Mesio-Buccal root canals.

It was observed that, in the BP direction, the taper increased the diameter along the length of the canal, F (4.94) = 4.152, p = 0.001, ƞ2 = 0.88, with an observed power of 95.3%. When comparing 1 M and 2 M, the latter showed a greater mean increase in taper (p = 0.001 [95% CI 2.50, 9.59]) than the 1 M. In the 1 M, the tapers remained constant (p > 0.05) until D4–5 (p = 0.013 [95% CI 1.38, 19.14]), where they decreased, remaining constant until D6–7 (p > 0.05). Unlike the 2 M, the taper varied significantly only on D5–6 (p = 0.025 [95% CI 1.43, 33.32]).

On the other hand, in the MD direction, the taper remained constant along the length of the canal, F (4.74) = 0.777, p = 0.561, ƞ2 = 0.561, with an observed power of 26.9%. In addition, it was observed that the mean taper in the 1 M was higher than in the 2 M (p = 0.029 [95% CI 0.19, 3.37]).

Disto-buccal root canals

In the BP direction, the taper remained constant along the length of the canal, F (3.20) = 0.664, p = 0.585, ƞ2 = 0.18, with a power of 19.1%. When comparing 1 M and 2 M, the latter showed greater taper than 1 M (p = 0.030 [95% CI 0.52, 9.33]). Similarly, in the MD direction, the taper remained constant throughout the length of the canal, F (3.78) = 1.33, p = 0.264, ƞ2 = 0.035, with a power of 40.5%. In addition, it was observed that the mean taper in the 2 M was higher than in the 1 M (p = 0.028 [95% CI 0.22, 3.64]).

Palatal root canals

It was observed in the BP direction how the means of conicity presented varied along the length of the canal without presenting any statistically significant difference, F (3,24) = 1.29, p = 0.279, ƞ2 = 0.29 with an observed power of 35.4%. When comparing 1 M and 2 M, they did not show statistically significant differences between their tapers (p = 0.781 [95% CI -3.29, 2.49]). In both molars, the greatest variability was observed in D5-6 (p = 0.064 [95% CI 2.73, 10.31]) and (p = 0.63 [95% CI -5.55, 11.84]), respectively.

On the other hand, in the MD direction, the conicity varied along the length of the canal, F (4.93) = 0.3.652, p = 0.004, ƞ2 = 0.77, with a power of 92.1%. The mean taper in the 2 M was greater than in the 1 M (p < 0.05 [95% CI 17.58, 22.69]).

The greatest variability in group 1 M in canal taper was observed between Sects. "Materials and methods" to "Results" (p = 0.097 [95% CI: 3.20; 13.67]) and 5 to 6 (p = 0.018 [95% CI: 3.45; 12.50]). In group 2 M, a slight reduction in taper was noted in Sects. "Conclusion" to 6 (p = 0.578 [95% CI: 3.748; 17.719]).

### Diameter comparison

The results of the Intraclass Correlation Coefficient (ICC) test, that reflects the level of agreement or reliability between diameter of root canals and files systems, with values ranging from 0 to 1, are shown in Table 3. Based on the ANOVA results an excellent compatibility was found in the MD direction of the MB and DB root canals (p > 0.05) in the Endogal® and Mtwo® systems (Table 3).

**Table 3:** Evaluation of the compatibility of diameters with different rotary systems

	Root canal		Rotary System													Effect size
			Endogal®				Protaper universal®			Mtwo®				Protaper Next®		
			EK1 (4%)	EK2 (6%)	EK3 (4%)	EK4 (4%)	S2	F1 (~ 7%)	F2(~ 8%)	0.15 (5%)	0.20 (6%)	0.25 (6%)	0.30 (4%)	X1 (~ 6%)	X2 (~ 6%)	
1 M	MB	BP	0.05	0.16	0.06	0.08	0.05	0.11	0.1	0.05	0.09	0.08	0.05	0.06	0.10	0.72
		MD	0.32	0.65*	0.57*	0.3	0.3	0.55	0.51	0.3	0.47	0.49*	0.3	0.39	0.53*	0.55
	DB	BP	0.09	0.15	0.1	0.13	0.03	0.16	0.16	0.09	0.13	0.15	0.07	0.10	0.16	0.69
		MD	0.56*	0.65*	0.54*	0.26	0.33	0.54*	0.49	0.4	0.51*	0.49	0.3	0.44	0.66*	0.62
	P	BP	0.12	0.2	0.11	0.30	0.1	0.23	0.23	0.12	0.18	0.2	0.1	0.14	0.35	0.63
		MD	0.07	0.11	0.07	0.16	0.06	0.13	0.12	0.07	0.1	0.11	0.05	0.08	0.11	0.71
2 M	MB	BP	0.08	0.13	0.09	0.11	0.09	0.16	0.14	0.09	0.13	0.13	0.07	0.12	0.16	0.92
		MD	0.60*	0.50	0.45	0.33	0.45	0.55*	0.49	0.43	0.55*	0.50	0.38	0.52	0.53	0.56
	DB	BP	0.2	0.19	0.13	0.13	0.13	0.22	0.2	0.16	0.19	0.19	0.1	0.08	0.12	0.55
		MD	0.52*	0.60*	0.52*	0.35	0.37	0.62*	0.61*	0.40	0.43	0.60*	0.44	0.38	0.60*	0.59
	P	BP	0.1	0.17	0.12	0.27	0.09	0.2	0.2	0.1	0.15	0.17	0.15	0.12	0.19	0.71
		MD	0.07	0.11	0.07	0.16	0.06	0.12	0.12	0.07	0.1	0.11	0.09	0.08	0.12	0.70

^*^p-value > 0.05 ANOVA of repeated measures, power of 1.00

1 M: 1st primary molar; 2 M: 2nd primary molar

MB Mesio-buccal canal; DB: disto-buccal canal; P: Palatal canal

BP: Bucco-palatal direction; MD: mesio-distal direction

The percentage values (e.g., 4%, 6%) indicate the taper of each instrument. The symbol " ~ " denotes an approximate taper, as certain instruments (e.g., ProTaper Universal® F1 and F2) have variable taper along their active length. Approximate taper values are based on the manufacturer's provided specifications

## Discussion

The analysis of the effectiveness of rotary instrumentation in the permanent dentition has led to modifications in rotary systems to increase endodontic treatment effectiveness. However, this innovation and development has yet to include the primary dentition.

According to some investigations, instruments designed for the permanent dentition have shown similar efficacy in the primary dentition (Manker et al. 2020). Subsequently, the manufacture of instruments adapted to the root length and compatibility with the mouth opening capabilities of a paediatric patient has been promoted. However, the instrumentation compatibility with the taper of the primary canal has yet to be evaluated. For this reason, the present anatomical study tested the compatibility of various commercial rotary files for adults, such as Protaper universal®, Mtwo® and Protaper Next®, in addition to Endogal® which is specific for the primary dentition.

### Analysis methodology used

Previously, root canal anatomy in primary teeth was studied using periapical radiographs, which provided an accuracy of approximately 65% for working length determination (Basso et al. 2015). Adjusting X-ray angulation improved the detection of complex canals to 91.5% accuracy in vitro (Sun et al. 2016). More recently, CBCT has enhanced anatomical analysis with high precision in both mesio-distal and bucco-palatal directions (Ozcan et al. 2016; Yang et al. 2013; Zoremchhingi et al. 2005). However, micro-CT offers greater detail, allowing for the visualisation of accessory canals and micro-communications (Fumes et al. 2014; Neboda et al. 2018; Torres-Ramos et al. 2020; Wang et al. 2013; Ziya et al. 2019). CBCT demonstrated 92.6% sensitivity and 100% specificity in detecting small canals (Khademi et al. 2022).

### Mean root canal lengths

The present study used root canal length measurements to standardise the number of measurements per canal type. Previous studies have reported that first molars (1 M) tend to have mean root lengths ranging from 6.1 to 7.8 mm for the MB root, 6.1 to 7.3 mm for the DB root, and 6.7 to 7.7 mm for the P root. In second molars (2 M), reported lengths are generally higher, ranging from 7.2–8.5 mm (MB), 6.9–8.1 mm (DB), and 8.3–8.9 mm (P) (Datta et al. 2019; Zoremchhingi et al. 2005; Ozcan et al. 2016). The present study found slightly lower but comparable values: 6.57 ± 1.18 mm (MB), 5.74 ± 1.25 mm (DB), and 5.94 ± 0.99 mm (P) for the 1 M, and 7.31 ± 1.02 mm (MB), 6.61 ± 1.06 mm (DB), and 6.34 ± 0.97 mm (P) for the 2 M.

The results of the present investigation indicated that the diameters of the MB and DB root canals of primary maxillary molars varied in the BP and MD directions. The MD diameter was of a smaller calibre concerning the BP. This pattern provides a cross-sectional oval shape to the canal; characteristics that it shares with some permanent teeth, such as the mandibular incisors. Veloso et al. described how the diameter of the lower incisors in the MD direction was smaller in the coronal (1.01 ± 0.15), middle (0.56 ± 0.14) and apical (0.28 ± 0.1) thirds compared to the diameter in the BL direction (1.32 ± 0.22; 1.67 ± 0.26; 0.43 ± 0.13) (Veloso-Carvalho de Oliveira et al. 2014). This anatomical characteristic is one factor that hinders the removal of infected dentine from the root canal due to the narrowing of the canal in the coronal third that, leaves a convex zone in the middle third, which is difficult to access. (Veloso-Carvalho de Oliveira et al. 2014).

It should be noted that the difference between MD and BP diameters is a characteristic that has been studied infrequently in primary molars. Fumes and colleagues analysed the diameters of the root canals, differentiating between the largest and shortest dimension, but did not report whether these diameters were measured in the BP or MD direction (Fumes et al. 2014). However, their findings have reinforced the idea of the oval conformation of the primary root canals.

One study has been found in the literature on conicity analysis, this was in primary anterior teeth with average root length being 10 mm (Torres-Ramos et al. 2020). In the present investigation, unlike the study by Torres-Ramos et al., the apical contraction was taken as the initial diameter (D0) because it is believed to be the area where there should be a maximum adaptation to the diameter of the rotary instrument so that it does not produce unwanted adverse events. On the other hand, measuring and determining the diameter in D0 assists in the selection of the ideal working instrument not to deform or transport the apex.

The measurements of the present investigation determined diameters in the MD direction in 1 M of 0.42 ± 0.33 for the MB root and 0.42 ± 0.2 for the DB root, whereas in 2 M the diameters were 0.23 ± 0.18 for the MB root and 0.26 ± 0.12 for the DB root. On the other hand, the palatal root at D0 for 1 M (0.38 ± 0.25) and 2 M (0.45 ± 0.24) tended to have a more circumferential conformation. Due to these results, we believe that the choice of the working instrument for primary molars should take into consideration a diameter at the tip of the ISO file between 0.20 and 0.40 mm. However, the choice of instrument will also depend on the curvature of the root and the compatibility along the entire length of the canal.

### Anatomical correlation

Based on the present results, all the rotary systems evaluated showed low compatibility in the BP direction. However, depending on the system used, some rotary files may present a moderate or substantial degree of compatibility in the MD direction.

The analysis of the compatibility of the rotary systems in 1 M root canals suggests a greater affinity the Endogal® systems (EK2 6% and Ek3 4%); Mtwo® (0.25 6%) and ProTaper Next® (X2) for the MB root. However, the Endogal® systems (EK2 6%); ProTaper Universal® (F1) and ProTaper Next® (X2) were more appropriate for the DB root.

Similarly, in terms of 2 M root canals, the rotary systems that presented the highest applicability for the MB root were Endogal® (EK1 4%), ProTaper Universal® (F1) and Mtwo® (0.20 6%), while the Endogal® systems (EK2 6%); ProTaper Universal® (F1); Mtwo® (0.25 6%) and ProTaper Next® (X2) were more appropriate for the DB root.

Concerning the palatal root of 1 M and 2 M, due to the irregularity of the diameters in the MD and BP directions and the significant variability of the taper, the systems analysed often had minor degrees of agreement. The greatest compatibility was in the BP direction, in 1 M with the EK4 files (CCI: 0.30); F2 (ICC: 0.23); X2 (CCI: 0.35) and in 2 M, the instruments that were most compatible were EK4 (CCI: 0.27) and X2 (CCI: 0.19).

### Strengths and limitations

The primary study strength was the significant number of diameters recorded along the canals, compared to previous investigations that only made three measurements (apical, mid, and cervical) (Barasuol et al. 2021).

The present research is the first of its kind as it sought to calculate the conicity of maxillary root canals by sectors and analyse their degree of anatomical compatibility with various rotary systems. We believe that this fact is a strength, as it opens a line of research for the technological development of instruments that allow better adaptation to the root structure of primary teeth, and at the same time, a limitation due to the lack of similar studies that allow comparison of results. Not considering the sex, age or race of origin of the collected teeth is another limitation since this could affect the morphological characteristics of the root canals.

## Conclusion

The root canals of primary maxillary molars presented a variable conicity. This variation in mesio-buccal and disto-buccal root canals is more accentuated in the BP direction, giving an oval shape to the canal. In palatal root canals, the taper between segments is highly variable.

The rotary instrumentation systems with the best adaptation to primary root canals were, in order, the Endogal® system, followed by the Protaper Universal® system; however, more studies are needed to confirm these results.

## Data Availability

The data that support the findings of this study are available from the corresponding author upon reasonable request.
